# Feeding Difficulties in Children with Hepatic Glycogen Storage Diseases Identified by a Brazilian Portuguese Validated Screening Tool

**DOI:** 10.3390/nu17111758

**Published:** 2025-05-23

**Authors:** Bárbara Cristina Pezzi Sartor, Bibiana Mello de Oliveira, Katia Irie Teruya, Lilia Ramos Farret, Tássia Tonon, Mariana Lima Scortegagna, Patrícia Barcellos Diniz, Carolina Fischinger Moura de Souza

**Affiliations:** 1 Hospital Materno Infantil Presidente Vargas, Porto Alegre 90035-074, Brazil; barbarapezzisartor@gmail.com (B.C.P.S.); patriciafono@icloud.com (P.B.D.); 2 Postgraduate Program in Genetics and Molecular Biology, Universidade Federal do Rio Grande do Sul, Porto Alegre 91509-900, Brazil; bmoliveira@hcpa.edu.br; 3 Postgraduate Program Psychology, Universidade Federal do Rio Grande do Sul, Porto Alegre 90035-002, Brazil; teruya.katia@gmail.com; 4 Medical Genetics Service, Hospital de Clínicas de Porto Alegre, Porto Alegre 90035-903, Brazil; lrefosco@hcpa.edu.br; 5 Postgraduate Program in Medical Sciences Medicine, Universidade Federal do Rio Grande do Sul, Porto Alegre 90035-003, Brazil; tassitonon@gmail.com (T.T.); scortegagnamariana@gmail.com (M.L.S.); 6 Postgraduate Program in Child and Adolescent Health, Universidade Federal do Rio Grande do Sul, Porto Alegre 90035-003, Brazil; 7 Clinical Research Service, Hospital de Clínicas de Porto Alegre, Porto Alegre 90035-003, Brazil

**Keywords:** glycogen storage disease, feeding and eating disorders, parent–child relations, psychological distress

## Abstract

**Background/Objectives**: Hepatic glycogen storage diseases (GSDs) are inherited metabolic disorders that affect glycogen synthesis or breakdown, primarily involving the liver and muscles. Treatment typically consists of strict dietary management, including the consumption of uncooked cornstarch. However, there is limited research on feeding challenges and the associated stress experienced by parents of children with GSDs. This study aims to assess feeding difficulties in children with GSDs and the level of parental stress. **Methods**: A total of 29 caregivers of children aged 6 months to <7 years participated. Feeding difficulties were evaluated using the Brazilian Infant Feeding Scale (*Escala Brasileira de Alimentação Infantil*—EBAI), while parental stress was measured using the Parental Stress Scale (*Escala de Estresse Parental*—EEPa). Data were collected in 2020, and the study was approved by the ethics committee. **Results**: The majority of the children were male (19/10), with a mean age of 47.75 months and an average age of diagnosis of 8.39 months. GSD type Ia (n = 15) and type Ib (n = 5) were the most prevalent, followed by types III and IX (n = 2). Among the participants, 22 out of 29 (76%) reported feeding difficulties, categorized as mild (n = 7, 24%), moderate (n = 7, 24%), and severe (n = 8, 28%). EBAI scores were higher in female patients and in those who did not eat meals with their family. Only one caregiver exhibited high levels of parental stress, as measured by the EEPA scale. No significant correlation was found between feeding difficulties and parental stress. **Conclusions**: The findings confirm a high prevalence of feeding issues in children with GSDs, which significantly affects caregivers’ quality of life. Although no significant link between feeding difficulties and parental stress was identified, further research is needed to improve GSD management and provide better support for caregivers.

## 1. Introduction

Hepatic glycogen storage diseases (GSDs) are rare genetic diseases belonging to the group of inborn errors of metabolism [1] that result in an alteration in glycogen metabolism, predominantly affecting the liver and muscles [2,3].

GSDs are classified according to enzyme deficiency and are classified into types: 0, I (Ia and Ib), III (IIIa and IIIb), IV, VI, IX (IXa, IXb, IXc), and XI [3,4]. GSD types IIIa, IV, IXb, and XI affect both liver and skeletal muscle. GSDs with predominantly hepatic involvement may present symptoms such as hypoglycemia, failure to thrive, hepatomegaly, increased liver enzymes, hyperlipidemia, and hepatic adenomas [5]. In addition, limb and trunk weakness, renal failure, cardiomyopathy, and osteopenia may be seen in GSDs with muscle involvement [6].

Adequate treatment reduces the risk of metabolic decompensation and ensures an excellent clinical prognosis. The use of uncooked cornstarch (UCCS) is widely used in patients with fasting intolerance [5,7], since it has the advantage of being neuroprotective when hypoglycemia occurs [8]. Previous studies showed that UCCS led to improvement in the management of these patients, with a good metabolic control and catch-up growth [9]. In GSD I, the restriction of simple sugars (maltose, fructose, lactose, galactose) and lactose-free formula are used [7,10]. For the ketotic forms of GSDs (types 0, III, VI, IX, and XI), a diet high in protein and low in simple carbohydrates is prescribed [6]. Additional strategies for treatment, such as the continuous infusion of glucose at night, ketogenic diet (for ketotic forms), medium-chain triglycerides (MCT), liver transplant, and gene therapy are described [11,12]. In some cases, where oral intake is insufficient or complicated by feeding difficulties, alternative nutritional routes such as nasogastric (NG) tubes or gastrostomy (G-tube) are employed to ensure adequate caloric intake [7].

Eating fulfills nutritional needs and serves as a cornerstone of socialization [13], especially after the introduction of solid foods at 4–6 months of age, when the child begins to be exposed to different textures and flavors, which, in the long term, helps to develop healthy behaviors [14]. For patients with GSDs, the goal is the same; however, the dietary restriction imposed by treatment, in addition to compromising micronutrient intake, can promote social isolation [6]. Furthermore, GSD patients more often display a selective food intake and increased food neophobia. It seems that patients, especially GSD I patients, may be at a higher risk of developing feeding disorders, as some patients even demonstrate complete food refusal [15]. When parents or caregivers take on the care of a child with feeding difficulties and seek to encourage variation in food supply and intake, family dynamics can be affected, both by the frustration generated by the breach of expectations and by the stress involved in the situation [16]. In addition, due to the high association with morbidity and mortality, caregivers feel pressured to provide the required amount of dietary intake and UCCS administration. However, pressure contributes to decreased intake and may promote negative responses to food [17,18]. The act of eating depends on the integration of motor and sensory systems [19,20]. Feeding promotes the development of relationships between children and parents, which is the basis for creating emotional bonds [21]. Considering the complexity and all the structures involved in this process, Goday et al. [22] defined food difficulties as a disorder in the oral intake of nutrients, incongruous with the individual’s developmental stage, persisting for a minimum duration of two weeks, and concomitant with medical, nutritional, alimentary competence, or psychosocial dysfunctions.

Feeding difficulties in at-risk populations can correspond to up to 80% of patients [23] and among their causes are gastrointestinal and cardiorespiratory diseases, sensory impairments, inadequate family habits, and behavioral and psychosocial issues [17,24,25,26]. Many of these characteristics are found in patients with hepatic GSDs, making them susceptible to developing feeding difficulties [25]. In one study, feeding difficulties were investigated in patients with hepatic GSDs, and 72.2% presented feeding difficulties [25]. However, no published study to date has used a validated scale for assessing feeding difficulties, but only their presence. In summary, feeding difficulties can affect the child’s development and impact the relationship between parents/caregivers and the child [20,21,22,23], i.e., parental stress is a factor to be considered when thinking about overall family well-being [27,28,29,30,31].

Within this framework, this study endeavors to examine the prevalence and severity of feeding difficulties by employing a validated scale designed for the Brazilian population. These assessments rely on the perspectives of parents and caregivers of children diagnosed with hepatic GSDs.

## 2. Materials and Methods

This research was a cross-sectional, descriptive, and quantitative study. It was submitted to and approved by the Ethics Committee on Human Research of the Presidente Vargas Maternal and Child Hospital (HMIPV-IRB), under number 4,342,128. All participants agreed to participate through the signing of an informed consent form.

**Figure 1:**
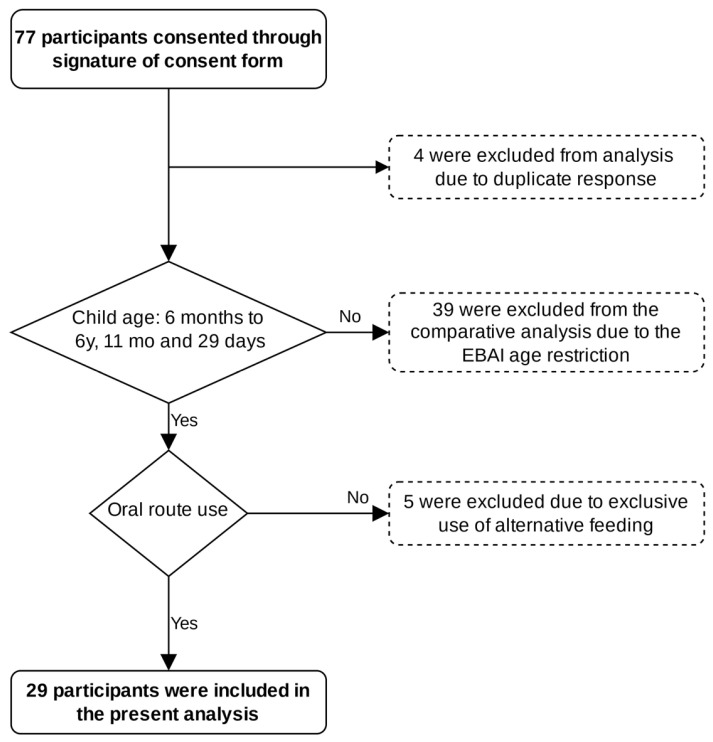
Flowchart of participant inclusion in this study.

The sample of this study consisted of 29 parents/caregivers responsible for the feeding of children diagnosed with hepatic GSDs. The inclusion criteria were being a parent/caregiver of children between 6 months and 6 years, 11 months, and 29 days old, diagnosed with hepatic GSDs, and receiving some form of oral feeding. The inclusion flowchart is illustrated in Figure 1.

The research was conducted online, and a virtual form in Brazilian Portuguese—created using the Google Forms platform—was distributed through the Brazilian Association of Glycogen Storage Diseases (*Associação Brasileira de Glicogenoses, ABGlico*). A snowball sampling method was adopted, allowing the form to reach a wider group of parents and caregivers of patients with hepatic GSDs from different treatment centers across the country. Parents/caregivers should complete an interview and fill out the Brazilian Scale of Infant Feeding (*Escala Brasileira de Alimentação Infantil*—EBAI) and the Parental Stress Scale (*Escala de Estresse Parental*—EEPa). Given the exploratory nature of the study and the sample size, descriptive and bivariate analyses were prioritized to observe general trends.

The EBAI is a self-report questionnaire consisting of 14 items and is interpreted by the T-score result, classifying difficulties as mild, moderate, and severe, presenting feeding difficulties when the T-score is greater than 60 [23,32]. Participants included in the study were required to be within the age range of 6 months to 6 years and 11 months, as the EBAI is a validated tool specifically designed for this age group [32]. At the time of data collection, no validated instrument was available for older children with similar feeding concerns. Although clear inclusion criteria were provided, the survey was self-administered by parents or caregivers—laypersons who may not have fully adhered to the written instructions. As a result, some responses fell outside the intended age range. These cases were excluded from the analysis to ensure methodological consistency and compliance with the tool’s validated parameters.

The EEPa is a questionnaire that can be self-administered, easy to handle, and free, consisting of 16 items, and the final score is produced by the sum of the items ranging from 0 to 64 points. The higher the score, the higher the parental stress [33]. However, this scale has not been normalized, meaning there are no studies with standardized cutoff points for the scale. In the study, the authors chose the following scheme as the cutoff point: scores above 50% were considered high parental stress and below 50% as low parental stress. As the EEPa is not a standardized instrument with established clinical thresholds for the Brazilian population—or specifically for caregivers of children with GSDs—this cutoff was used as a pragmatic criterion to allow for exploratory categorization and preliminary group comparisons. It was adapted and validated for Brazilian Portuguese, with the author of the validation authorizing its application. The caregivers of the children received the questionnaires online and answered them individually and without intervention, with the possibility of clarifying doubts.

The study data were tabulated in Excel software and the analyses were performed in the SPSS program (v. 21.0), with a significance level of 5% adopted. Quantitative variables were described by mean and standard deviation or median and interquartile range. Categorical variables were described by absolute and relative frequencies. To compare means, t-Student tests or one-way analysis of variance (ANOVA) were applied. In case of asymmetry, Mann–Whitney or Kruskal–Wallis tests were used. In the comparison of proportions, Fisher’s exact test was applied. To evaluate the association between numerical and ordinal variables, Pearson’s linear correlation tests or Spearman’s were used, respectively.

## 3. Results

In this study, a total of 29 participants, comprising parents and/or caregivers of individuals with hepatic GSDs, were included. The patients under their supervision exhibited a predominant male demographic (M:19; F:10). The average age of these patients at the time of their participation was 47.75 months, with an age range spanning from 14 to 78 months. Furthermore, the average age at which the hepatic GSD was diagnosed in this cohort was 8.39 months, ranging from 0 to 48 months.

Regarding the type of hepatic GSD, type 1a emerged as the predominant subtype, as reported by 15 individuals. Four participants could not provide specific information about the type of hepatic GSD they had.

A comprehensive overview of the sample demographics is presented in Table 1.

In the context of feeding routes, seven children (24.1%) necessitated an alternative approach. The primary alternative route employed was gastrostomy (n = 6, 85.7%). The mean age of gastrostomy insertion was 13.1 months (range: 4 to 48) (Table 2). It is noteworthy that only patients diagnosed with GSD types 1a (n = 6) and 1b (n = 1) required an alternative route.

The EBAI scale presented a mean T-score of 65.8 ±10.3 (range: 41 to 87), indicating the presence of feeding problems (score > 60) in 22 participants (76.0%). Eight participants (28.0%) presented severe feeding difficulties. Within the sample, seven participants (24.0%) had no feeding problems, seven experienced mild difficulties (24.0%), seven faced moderate difficulties (24.0%), and eight had severe difficulties (28.0%).

The mean score on the EEPA scale was 11.9 ± 8.3 points (range: 0 to 41). Only one parent/caregiver had high parental stress (scoring 41 on the EEPA).

It was observed that the EBAI score was significantly higher among female patients. Patients who did not have meals with their families had a higher EBAI score. Individuals with a history of nausea or signs of discomfort with the smell, touch, and sight of food had a significantly higher EBAI T-score (Table 3).

**Figure 2:**
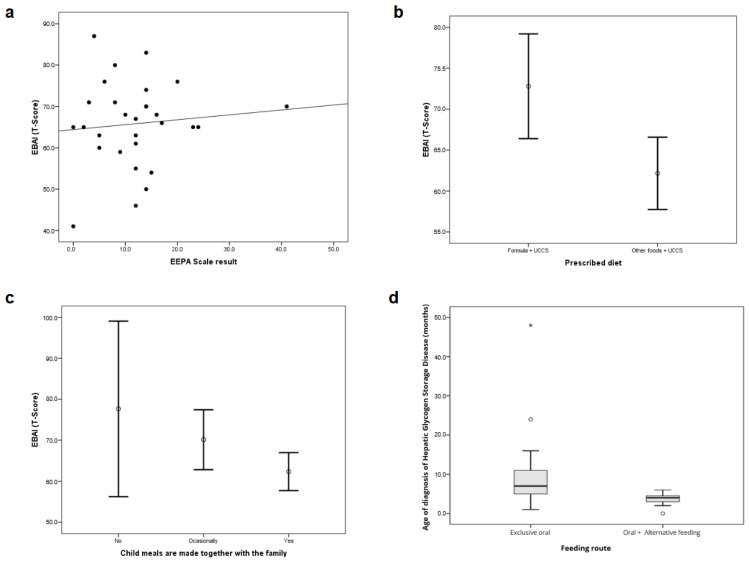
(**a**) Comparison between the results of EBAI and EEPa scales (Spearman correlation). (**b**) Comparison between EBAI results and prescribed diet (t-Student test). (**c**) Comparison between EBAI scale results and whether the child has meals with the family (t-Student test). (**d**) Comparison of age at diagnosis of hepatic GSD with feeding route. Asterisks in (**d**) indicate outlier data points.

There was no correlation observed between the scores on the EBAI and EEPa scales (rs = 0.075; *p* = 0.701) using the Spearman correlation coefficient (Figure 2a).

Nevertheless, the EBAI T-score was correlated with both the prescribed diet (*p* = 0.006; t-Student test) (Figure 2b) and whether the child had meals with the family (*p* = 0.019; one-way ANOVA) (Figure 2c). These findings underscore the influence of both dietary compliance and family mealtime context on eating behavior in children with hepatic GSDs.

The feeding method showed a statistically significant correlation with the age of diagnosis of hepatic GSD (*p* = 0.008; Mann–Whitney test) (Figure 2d), suggesting that earlier diagnosis may impact the need for more structured or assisted feeding strategies. Together, these results provide insights into the longitudinal effects of tailored nutritional management on the clinical trajectory of patients with hepatic GSDs.

## 4. Discussion

This study represents the inaugural assessment of feeding challenges and parental stress in parents/caregivers of children with hepatic GSDs through the utilization of validated scales, specifically the Brazilian Portuguese versions of the EBAI and EEPa.

Regarding the EBAI, twenty-two individuals (76%) exhibited manifestations of feeding difficulties, a finding that aligns with prior research demonstrating feeding challenges in high-risk populations (approximately 80%) and corroborates a previous investigation on patients with hepatic GSDs, which reported a prevalence of 72.2% in feeding difficulties [23,24,25]. Nonetheless, it is worth noting that no statistically significant correlation was observed between the EBAI and EEPa scales.

The EBAI demonstrated a significant correlation with the prescribed diet, revealing that individuals who exclusively consumed a lactose-free formula along with MCT exhibited higher T-scores on the EBAI. This outcome suggests an increased level of feeding difficulty in patients adhering to a restricted dietary regimen. It should be emphasized that among these patients, 60% were reliant on exclusive oral feeding, with only 10% exhibiting no discernible alterations in the EBAI. Therefore, children exhibiting heightened feeding challenges, as indicated by these findings, were primarily those for whom feeding served a functional role, primarily centered around nutritional and therapeutic aspects, with limited emphasis on responsive feeding strategies that foster a symbiotic relationship with food. Such responsive feeding involves emotional connections and is inherently linked to the process of learning, contingent upon temporal factors, experiences, opportunities, comfort, role models, and the cultivation of a health-promoting environment [17,18,20,34].

The EBAI also found a notable correlation between children who did not share meals with their families and higher scores, indicative of more significant feeding difficulties [35]. Previous research has consistently underscored the health benefits of family meals, including a more nutrient-rich diet, improved dietary balance, and a decreased risk of developing eating disorders and substance abuse problems such as alcohol and drug abuse. Additionally, communal meals promote a greater appreciation of food and can lower the likelihood of feeding difficulties [36,37].

Likewise, in individuals with a history of nausea and/or discomfort with the smell and/or touch and/or sight of food, the EBAI T-score was significantly higher, indicating that feeding difficulties go beyond and begin before food intake. This result reinforces the fact that among feeding alterations, there are those resulting from an altered sensory system [38,39,40], causing discomfort in the individual, who reacts negatively to stimuli due to the accumulation of information sent to the central nervous system [38,40,41].

There was a higher prevalence of the use of alternative feeding routes, including those complementary to oral feeding, among patients with an early disease diagnosis. This observation implies a potentially reduced exposure to oral feeding pressure in these children, as a part of their diet and/or treatment is delivered via alternative routes—NG, NE, or G-tube. However, previous studies, such as Bérat et al., have shown that after G-tube, children with GSDs (n = 23) presented eating problems (but an improvement in quality of life) [42]. Also, there is an association between food refusal and NG exposure > 3 months in children with various disorders [43]. Therefore, it can be expected that patients with GSDs may experience eating disorders as well as psychosocial problems after receiving a feeding tube or G-tube. Refusal to eat can lead to life-threatening situations for these patients with fasting intolerance, causing stress for both the patient and the parents, possibly worsening eating and psychosocial problems [15]. In addition, food refusal combined with the monotony of the allowed diet impairs the intake of micronutrients important for the child’s growth and development. Nutritional deficiencies related to B vitamins have already been described in patients with GSDs and the prescription of dietary supplements to achieve the daily recommendation is encouraged [44]. In our study, four (57.2%) of the seven children who use NG, NE, or G-tube receive only lactose-free formula through one of these routes, emphasizing that feeding is an essential learning process in the development of one’s relationship with food [20,23].

In the present sample, males predominated, whereas in two other studies, females were more prevalent [7,45]. One of these studies referenced the International Registry of Severe Chronic Neutropenia, which stores data from 37 patients with GSD Ib: 23 adults (16 females and 7 males) and 14 children (9 females and 5 males) [7]. However, the findings align with those of Martinez et al. [24], where the sample of 36 individuals consisted of 19 males and 17 females.

This study has several limitations. Data were collected exclusively via online self-report questionnaires, which may introduce response or comprehension biases. The identity of the caregiver respondent (e.g., mother, father, both) was not specified, limiting the interpretation of stress and feeding perceptions.

The EEPA scale does not differentiate whether parental stress is feeding-specific or related to other caregiving aspects, nor does it categorize stress levels—only indicating that higher scores reflect greater stress. Additionally, the presence of negatively framed items may have caused discomfort, despite the anonymity of responses.

Regarding the validity and sensitivity of the EEPa and EBAI scales for caregivers of individuals with GSD, it is important to note that, although both instruments have shown reliability and validity in general populations, they have not been specifically validated for this clinical group. Thus, their use in this context should be interpreted as exploratory. Future research should aim to assess the psychometric performance of these tools within rare disease populations to ensure their appropriateness and sensitivity to the unique stressors these caregivers face.

Selection bias is possible, as participation required digital access and may have favored families more connected to patient associations. The accuracy of reported GSD subtypes may also be affected, as diagnoses were self-reported by caregivers.

Finally, the cross-sectional design and limited sample size restrict the generalizability of the findings and preclude any longitudinal analysis. Future studies should consider combining targeted instruments with mixed-methods approaches—such as qualitative interviews and follow-up assessments—to better identify the specific sources of parental stress and clarify the relationship between stress and feeding difficulties in this population. In addition, longitudinal tracking of feeding development, as well as the implementation and evaluation of interventions targeting sensory sensitivity or other contributing factors, may provide deeper insight and support more tailored, effective care strategies for children with GSDs and their families.

The findings of this study have strong translational relevance for healthcare professionals, particularly dietitians, pediatricians, and multidisciplinary teams working with children with GSDs. Understanding the feeding difficulties and parental stress associated with GSDs allows these professionals to tailor interventions that address not only the medical aspects of the disease but also the social and emotional well-being of the child and their family. A family-centered, preventive, and responsive approach, informed by the insights gained from this study, can help optimize nutritional management and improve quality of life by fostering better outcomes in both dietary adherence and stress reduction for parents and caregivers.

Overall, parental stress levels in the cohort were relatively low, which may reflect adequate psychosocial support, adaptation to the care routine, or effective coping mechanisms. However, the presence of a single high score underscores the need for individualized psychosocial screening, as isolated cases of elevated stress may go unnoticed without systematic assessment.

The innovation and significance of this study are underscored by its utilization of quantitative measures to screen variables: feeding difficulties and parental stress in patients with hepatic GSDs. Given the substantial impact that feeding difficulties can have on daily life and social participation, early identification and family-centered treatment approaches are essential. These should prioritize functional and meaningful outcomes to enhance the health and quality of life for both children and their caregivers [46,47,48,49].

Therefore, further research is needed to deepen the understanding of this topic. Future studies could include longitudinal monitoring of children’s feeding development to observe changes over time. Additionally, it would be relevant to utilize specific interventions, such as responsive feeding therapy, which aims to support parents in understanding their children’s needs and to help children through responsive food exposures and the exploration of intrinsic motivation, facilitating new connections during mealtimes [50].

Importantly, this is the first study to explore these variables using validated tools specifically adapted to Brazilian Portuguese (EBAI and EEPa), allowing for cultural and linguistically appropriate assessment. This novel approach not only advances the understanding of the psychosocial dimensions of hepatic GSDs, but also sets a foundation for future interventions and cross-cultural research in this area.

## 5. Conclusions

This study represents a novel contribution to the field by employing validated assessment tools in Brazilian Portuguese—specifically, the Brazilian Scale of Infant Feeding (EBAI) and the Parental Stress Scale (EEPa)—to quantitatively evaluate feeding difficulties and parental stress in patients with hepatic GSDs. By doing so, it provides objective data on the prevalence and severity of feeding challenges, which were consistent with findings in other at-risk populations (approximately 80%) and aligned with a previous report on hepatic GSD patients (72.2%). Although the scales used were not statistically correlated, quantifying parental stress was instrumental in understanding how a chronic diagnosis such as GSD can affect family quality of life.

In conclusion, this study highlights the importance of using culturally and linguistically validated instruments to better capture the lived experiences of families. Future research should further explore dietary management complexities and the psychosocial stressors experienced by caregivers, with the goal of improving both patient and family outcomes.

## Figures and Tables

**Table 1:** Sample characterization.

Variables	n = 29
Age (months)—Mean ± SD [range]	49.7 ± 19.9 (14–79)
Gender—n (%)	
Male	19 (65.5)
Female	10 (34.5)
GSD Type—n (%)	
Ia	15 (51.7)
Ib	5 (17.2)
III	2 (6.9)
VI	1 (3.4)
IX	2 (6.9)
Unknown	4 (13.8)
Family Income (in minimum wages)—n (%**)**	
<1	10 (34.5)
1 to 2	4 (13.8)
2 to 3	7 (24.1)
3 to 5	3 (10.3)
5 to 10	1 (3.4)
>10	4 (13.8)

Abbreviations: GSD = hepatic glycogen storage disease.

**Table 2:** Sample characterization regarding diet and feeding route.

		n	%
Prescribed diet	Foods + UCCS	19	65.5
	Lactose-free formula + UCCS	10	34.5
Feeding method	Exclusive oral	22	75.9
	Oral + alternative feeding method	7	24.1
Alternative feeding method	Gastrostomy	6	20.7
	Nasogastric or nasoenteral tube	1	3.4
Place of meals	At the table	19	65.5
	Other (living room, bedroom, kitchen)	10	34.5
Family meals	Yes	19	65.5
	No	3	10.3
	Sometimes	7	24.2
Nausea or discomfort when looking at the food		15	51.7
Nausea or discomfort when touching the food		15	51.7
Nausea or discomfort related to the smell of the food		18	62

Abbreviations: UCCS = uncooked cornstarch.

**Table 3:** Scores on the EBAI and EEPa scales according to sex and treatment-related variables.

		EBAI Results (T-Score)			EEPA Results	
		Mean	Standard Deviation	*p*	Median (P25–P75)	*p*
Child biological sex	Female	71.1	9.2	0.043 *	10.5 (5.5–18)	0.982
	Male	63.1	10.0		12 (5–14)	
Family income (in minimum wage)	<1	67.2	13.3	0.940	14 (11–16)	0.059
	1 to 2	67.8	10.4		13 (7–21)	
	2 to 3	64.3	10.6		3 (0–12)	
	3 to 5	60.7	5.9		15 (5–23)	
	5 to 10	70.0	0		41 (41–41)	
	>10	66.0	7.7		9.5 (7–12)	
Prescribed diet	Only lactose-free formula + MCT oil	72.8	8.9	0.006 *	8.5 (6–14)	0.211
	Foods + UCCS	62.2	9.2		12 (5–17)	
Feeding method	Oral + alternative feeding method	68.7	9.6	0.405	14 (8–20)	0.304
	Exclusive oral	64.9	10.6		12 (5–14)	
Alternative feeding method	Gastrostomy	69.3	10.4	0.716	13 (7–16)	0.286
	NG/NE	65	0.0		24 (24–24)	
Place of the meals	At the table	64.5	6.9	0.467	12 (6–14)	0.839
	Others	68.3	15.0		13 (3–21)	
Family meals	Yes	62.4	9.6	0.019 *	12 (6–14)	0.558
	Sometimes	70.1	7.9		10 (2–23)	
	No	77.7	8.6		20 (4–41)	
Nausea or discomfort from the smell of the food	Yes	69.5	10.0	0.011 *	11 (5–17)	0.774
	No	59.8	7.9		12 (9–14)	
Nausea or discomfort when looking at the food	Yes	71.6	7.1	0.001 *	12 (5–17)	0.621
	No	59.6	9.7		12 (6–14)	
Nausea or discomfort when touching the food	Yes	70.8	8.2	0.005 *	12 (5–17)	0.621
	No	60.5	9.8		12 (6–14)	

Abbreviations: MCT = medium-chain triglycerides; UCCS = uncooked cornstarch; NG = nasogastric tube; NE = nasoenteral tube. ANOVA and Mann–Whitney test, *: statistically significant.

## Data Availability

The raw data supporting the conclusions of this article will be made available by the authors on request.

## Abbreviations

The following abbreviations are used in this manuscript:

ABGlico	Associação Brasileira de Glicogenoses
ANOVA	Analysis of variance
EBAI	Brazilian Scale of Infant Feeding
EEPa	Parental Stress Scale
GSD	Hepatic glycogen storage disease
G-tube	Gastrostomy
HMIPV-IRB	Hospital Materno Infantil Presidente Vargas-
MCT	Medium-chain triglycerides
NE	Naso-enteral tube
NG	Nasogastric tube
UCCS	Uncooked cornstarch
